# Single Capsule Bismuth Quadruple Therapy for Eradication of *H. pylori* Infection: A Real-Life Study

**DOI:** 10.3389/fphar.2021.667584

**Published:** 2021-04-29

**Authors:** Antonietta G. Gravina, Kateryna Priadko, Lucia Granata, Angela Facchiano, Giuseppe Scidà, Rosa Cerbone, Paola Ciamarra, Marco Romano

**Affiliations:** Hepatogastroenterology Division, Department of Precision Medicine, University of Campania Luigi Vanvitelli, Naples, Italy

**Keywords:** BQT, bismuth quadruple therapy, *H. pylori*, real life, eradication therapy

## Abstract

**Background and aim:** Bismuth quadruple therapy (BQT) or non-bismuth quadruple therapy (i.e., concomitant therapy) (CT) is the first-line regimens to eradicate *H. pylori* infection in areas with high prevalence of clarithromycin (CLA) resistance. Guidelines suggest that in areas of high prevalence of *H. pylori* strains with double resistance (i.e., CLA + metronidazole), BQT should be preferred to CT. The aim of this study was to evaluate the efficacy and safety of BQT administered through the three-in-one pill (Pylera) formulation in a large series of *H. pylori*–infected patients, naive to treatment in a region with high CLA and dual resistance.

**Patients and methods:** We treated 250 patients (148 F and 102 M, mean age 48.6 years) with H. *pylori* infection naïve to treatment. Patients received esomeprazole 40 mg bid and Pylera 3 tablets qid for 10 days. Diagnosis of H. *pylori* infection was through ^13^C urea breath test (^13^C UBT), or stool antigen test or histology, as appropriate. The evaluation of eradication was through ^13^C UBT at least 45 days after the end of therapy. Incidence of treatment-related adverse events (TRAEs) was assessed through a questionnaire at the end of treatment. Compliance was considered good if at least 90% of medication had been taken. Statistical analysis was per intention-to-treat e per protocol (PP). 95% confidence intervals (CIs) were calculated.

**Results:** 1) 13 patients (5.2%) discontinued therapy due to side effects; 2) eradication rates in ITT and PP were 227/250 (90.8%; 95% CI 86.3–93.7%) and 226/237 (95.3%; 95% CI 91–99%), respectively; 3) the prevalence of TRAEs was 26.8%; and 4) adherence to treatment was good with compliance greater than 90%.

**Conclusion:** In this real-life study, we demonstrate that in an area with a high prevalence of *H. pylori* strains with CLA or CLA + metronidazole resistance, BQT using Pylera is an effective therapeutic strategy with ITT eradication rates higher than 90%; this therapy is associated with good compliance and low incidence of side effects.

## Introduction

*Helicobacter pylori* (*H. pylori*) is a Gram-negative microorganism isolated for the first time in 1982 (Warren and Marshall, 1983). Currently *H. pylori* infection has a worldwide prevalence of about 50%, with the highest prevalence in developing countries related to socioeconomic status within societies and hygiene habits and conditions (Hooi et al., 2017).

*H. pylori* infection is associated to a number of gastroduodenal pathologic conditions and also extragastric diseases (Kusters et al., 2006; Gravina et al., 2018; Gravina et al., 2020). Up-to-date*, H. pylori* is proved to be main the causative factor of chronic gastritis, peptic ulcer disease, gastric adenocarcinoma, and MALToma (Kusters et al., 2006). Also, dyspepsia associated to *H. pylori* infection is now regarded as an organic form of dyspepsia. The Italian Guidelines and Maastricht V/Florence Consensus recommend test-and-treat strategy in patients with dyspeptic symptoms under the age of 50 years without alarming signs (Zagari et al., 2015; Malfertheiner et al., 2017). H*. pylori* diagnosis should be based on urea breath test (^13^C) (UBT), monoclonal stool antigen test (SAT), or histology that bear high pre- and posttreatment diagnostic value, while positive serology (i.e., detectable serum levels of IgG against *H. pylori*) does not discriminate past vs. ongoing infection (Zagari et al., 2015; Malfertheiner et al., 2017).

The choice of eradication strategy is another challenging aspect in the management of *H. pylori* infection due to the increasing prevalence of *H. pylori* clinical isolates which are resistant to the antimicrobials currently used to treat the infection. In particular, there is the need of an efficient (i.e., with an eradication rate >90%) first-line empirical therapy because treatment failure leads to an increased prevalence of *H. pylori* resistant strains (Romano et al., 2008). According to Maastricht V/Florence Consensus, the first-line eradication therapy should be based on local prevalence of *H. pylori* strains resistant to clarithromycin (CLA). A country is defined as one with a high CLA resistance when CLA resistance is equal or higher than 15–20% (Malfertheiner et al., 2017). In countries with low resistance to CLA, a 10–14 days triple therapy should be used. On the other side, non-bismuth quadruple (i.e., concomitant) therapy or bismuth quadruple therapy (BQT) should be used in countries with high CLA resistance. Prevalence of *H. pylori* strains with dual resistance to CLA and metronidazole is increasing in many European countries, Italy in particular (De Francesco et al., 2006; Savoldi et al., 2018), thus making it more troublesome to eradicate the infection. While CLA-containing regimens such as concomitant, sequential, or bismuth quadruple therapies are the first-line regimens in regions with CLA resistance over 15%, BQT is the preferred eradication regimen in areas with a high prevalence of dual (CLA + metronidazole) resistance1 (Zagari et al., 2018; Malfertheiner et al., 2017). However, a recent study has demonstrated that concomitant therapy achieves eradication rates comparable to those obtained with BQT (Romano et al., 2020). A recent report based on data collected through the European Registry on *H. pylori* management (Hp-EuReg) on 21,533 patients in various European countries found out that only BQT lasting at least 10 days or optimized 14-day non-bismuth quadruple (i.e., concomitant) therapy is able to achieve eradication rates above 90% (Nyssen et al., 2021).

Besides bacterial resistance, failure of eradication therapy might at least in part be due to an inadequate increase in gastric pH during PPI therapy which leads to a decreased bioavailability of antimicrobials in the gastric lumen in Caucasian subjects in whom prevalence of extensive and intermediate metabolizers of PPI is as high as >95% (Shi and Klotz, 2008). Because of this, it has been suggested (Molina-Infante et al., 2013) to optimize the therapy by doubling the dose of PPI

This study was therefore designed to assess in a large series of patients collected in a single center whether optimized BQT showed high efficacy (i.e., eradication rate over 90%) in the real world, in a region with high CLA and dual resistance. As secondary outcomes, the prevalence of treatment-related adverse events (TRAEs) and compliance to treatment were evaluated

## Patients and Methods

**FIGURE 1:**
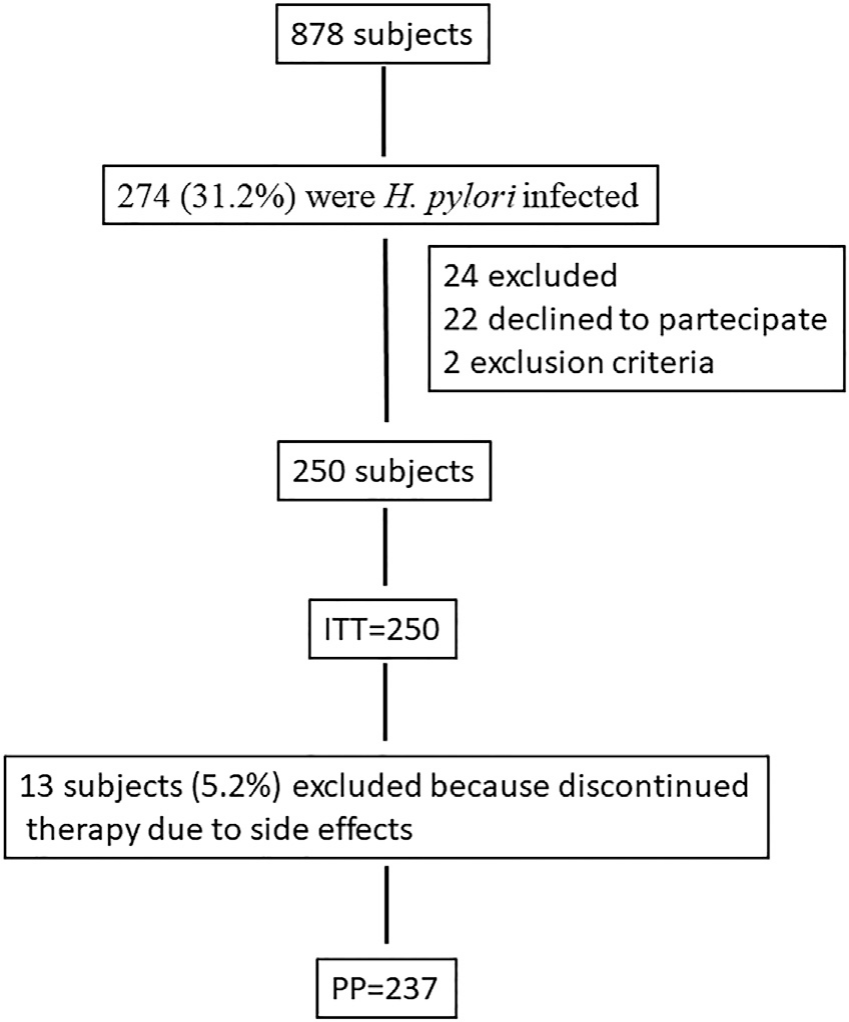
Flow diagram of the study.

This is a real-life study performed on consecutive patients referred to the Gastroenterology Unit of University of Campania “L. Vanvitelli” because of dyspeptic symptoms who tested positive for *H. pylori* infection and were naive to treatment from January 2018 to June 2020 (Figure 1). Inclusion criteria were as follows: age above 18 years; written informed consent to be enrolled into the study; no previous *H. pylori* eradication treatment; and last use of antibiotics at least four weeks prior to the treatment and/or use of acid suppressing drugs at least two weeks prior to treatment. *H. pylori* diagnosis was performed by ^13^C-urea breath test (^13^C UBT), stool antigen test (SAT), or histology in those patients who underwent esophagogastroduodenoscopy (EGDS). In particular**,** for ^13^C UBT a baseline breath sample was obtained, and 100 mg of ^13^C urea with citric acid (1.4 g) was administered as an aqueous solution (Expirobacter; SOFAR, Milano, Italy). Another breath sample was collected 30 min later. The test result was considered positive if the difference between the baseline sample and the 30-min sample exceeded 5.0 parts/1,000 of ^13^CO2 (Federico et al., 2012a). SAT was performed using monoclonal HpSA (HEPY Stool Card Plus, Mascia Brunelli SpA, Milano, Italy).

Assessment of *H. pylori* eradication following the treatment was performed 45 days after the end of treatment in order to minimize the likelihood of false negative results. ^13^C UBT was used in the vast majority of patients, whereas histology was used only in five patients in whom EGDS was required.

We used single capsule BQT (i.e., Pylera^Ⓡ^), approved by the Italian National Health System, for 10 days as an eradication regimen for *H. pylori* infection. Each Pylera capsule contains bismuth subcitrate 140 mg, metronidazole 125 mg, and tetracycline 125 mg. Therapy was as follows: esomeprazole 40 mg before breakfast and dinner + Pylera^Ⓡ^three capsules after breakfast, lunch, dinner, and bedtime. The incidence of TRAEs was evaluated via a questionnaire at the end of therapy. Compliance was considered to be good if patients took at least 90% of medications. Patients who did not eradicate the infection were retreated with a 14 days levofloxacin-containing quadruple therapy (i.e., esomeprazole 40 mg bid plus amoxicillin 1 b bid plus levofloxacin 250 mg bid plus tinidazole 500 mg bid)**.**

### Statistical Analysis

The primary efficacy variable was the eradication rate of *H. pylori*. We analyzed eradication rates in intention-to-treat (ITT) and per protocol (PP) analysis. ITT analysis included all enrolled subjects who took at least one medicine from the study protocol. PP analysis included only those subjects who strictly adhered to the protocol, received no less than 90% of medications, and underwent eradication confirmatory test that was presented at the follow-up visit. Patients who did not perform eradication test and/or were absent at the follow-up visit were considered lost to follow-up. We calculated mean with standard deviation (SD) for continuous variables and percentages with 95% confidence interval (CI) for categorical variables. A Student t test, chi-squared test, or Fisher exact test, as appropriate, was performed to compare demographic characteristics and eradication rates between treatment groups. A *p* value of <0.05 was considered statistically significant. All analyses were performed with STATA statistical software (StataCorp).

## Results

We screened 878 dyspeptic patients and 274 (31.2%) were *H. pylori* infected. Figure 1 shows the flow diagram of the study participants. Twenty-four were excluded from the study. A total of 250 patients (148 F and 102 M, mean age 48.6 years) were treated with three-in-one BQT. Table 1 shows the baseline characteristics of the patients. 13 patients discontinued therapy for side effects (13/250; 5.2%). One of these patients who took therapy for only 7 days, however, underwent ^13^C UBT, after 45 days from the end of therapy, with a negative result. We calculated eradication rates in ITT and PP analysis, and we obtained eradication in 227/250 (90.8%; 95% CI 86.3–93.7%) in ITT analysis and in 226/237 (95.3%; 95% CI 91–99%) in PP analysis. The prevalence of TRAEs was 26.8% (67/250). Also, 5.2% (13/250) of patients interrupted treatment because experiencing severe TRAE. Table 2 illustrates TRAE variety and rates. The compliance was good in 237/250 (94.8%) patients who took >90% of prescribed medicines. Ten patients who did not eradicate the infection were retreated with a levofloxacin-containing quadruple therapy and all of them resulted negative 45 days after the end of therapy.

**TABLE 1:** Baseline information about the study population.

Characteristic	Number (%)
Total subjects	250
Female gender	102 (40.8)
Mean age, years (SD)	48.6 (11.1)
Smoking	82 (32.8)
**Test pre-treatment**	
^13^C UBT	143 (57.2)
Histology	89 (35.6)
SAT	10 (4)
SAT + Histology	3 (1.2)
^13^C UBT + SAT	5 (2)
**Test posttreatment**	
^13^C UBT	233 (93.2)
Histology	5 (2)
None	12 (4.8)

**TABLE 2:** Treatment-related adverse events (TRAEs).

TRAEs	n (%)
Patients with TRAEs	67/250 (26.8)
Treatment discontinuation due to TRAEs	13/250 (5.2)
Gastrointestinal disorders	49 (19.6)
Nausea	23/250 (9.2)
Diarrhea	20/250 (8)
Abdominal pain	20/250 (8)
Dyspepsia	10/250 (4)
Vomiting	9/250 (3.6)
Central nervous system	29/250 (11.6)
Dysgeusia	23/250 (9.2)
Headache	11/250 (4.4)
Dizziness	9/250 (3.6)
Sonnolence	2/250 (0.8)
Others	
Asthenia	17/250 (6.8)
Cutaneous itch	3/250 (1.2)

## Discussion

Italy is an area with high prevalence of primary resistance toward CLA and metronidazole with a resistance to CLA equal to 30% and a double resistance to CLA and metronidazole as high as 19.3% (Fiorini et al., 2018). In this setting, most of the international guidelines suggest that BQT should be the preferred first-line option (Zagari et al., 2015; Malfertheiner et al., 2017). Recently, a three-in-one pill formulation has been used in place of the conventional BQT, offering the possibility of combining in one pill all of the antimicrobials used in the standard BQT (Di Ciaula et al., 2017; Gómez Rodríguez et al., 2017; Tursi et al., 2017). This formulation has shown to be effective in the treatment of *H. pylori* infection achieving eradication rates ranging from 93 to 98% (Tursi et al., 2017; Gómez Rodríguez et al., 2017). In our single center, real-life study in 250 *H. pylori*–infected patients naive to treatment, we confirm that BQT administered through the novel three-in-one-pill formulation is a highly effective eradication regimen in an area with high prevalence of CLA or double resistance to CLA and metronidazole (Tursi et al., 2017). Eradication rates in ITT analysis were 90.8% (95% CI 86.3–93.7%) and in PP analysis 95.3% (95% CI 91–99%). Our results are therefore similar to those obtained in other studies but on a greater number of patients and in a real-life clinical setting (Romano et al., 2020; Zagari et al., 2018).

Side effects may affect compliance to and efficacy of treatment. In this study, we show that single capsule BQT is associated with good compliance and low incidence of side effects. In our study, compliance was greater than 90% and did not seem to be influenced by the high number of pills which patients had to take daily. Also incidence of TRAEs was of about 26% and only a small percentage of patients (i.e., 5%) discontinued treatment because of severe side effects. This is also in agreement with previous studies (Romano et al., 2020; Fiorini et al., 2018; Gómez Rodríguez et al., 2017; Tursi et al., 2017; Zagari et al., 2018).

Levofloxacin-containing triple therapy has shown efficacy in patients naive to treatment (Federico et al., 2012b). Moreover, Gisbert et al. (2014) showed that levofloxacin-containing eradication regimens are useful as second- or third-line treatment, in particular if bismuth is added. In many countries, such as Italy, bismuth compounds are no longer available, and, therefore, their use in eradication regimens is not possible. According to the recommendations of most international guidelines, we decided to treat patients who failed to eradicate the infection with BQT, using a levofloxacin-containing regimen and decided to increase the efficacy of levofloxacin-containing triple therapy (i.e., the recommended therapy after BQT failure) by adding tinidazole. Although in a small number of subjects, we here demonstrate that a non-bismuth levofloxacin-containing quadruple therapy is highly effective as second-line treatment after failure to eradicate the infection with Pylera. In fact, all of the ten patients who were still positive after single capsule BQT were eradicated of the infection. A study with a larger number of subjects is however necessary to corroborate this result.

The use of PPIs in the eradication of *H. pylori* has demonstrated to increase the eradication rate (Calvet and Gomollón, 2005; Gomollón and Calvet, 2005; McKeage et al, 2008; Baldwin and Keam, 2009; Kirchheiner et al, 2009; McNicholl et al., 2012) by increasing the stability of antibiotics in a less acidic gastric environment thus inducing a higher antibiotic concentration and antibacterial efficacy (McNicholl et al., 2012; Calvet and Gomollón, 2005). Many studies in humans have shown that differences on acid control account for differences in eradication rates and that strong acid inhibition increases the efficacy of *H. pylori* treatments (Boparai et al., 2008; Gisbert et al., 2003). Therefore, in our clinical setting, we decided to optimize therapy by doubling the daily dose of esomeprazole (i.e., 40 mg bid) as already successfully done in previous studies (Romano et al., 2020; McNicholl et al., 2012).

This study has some limitations. First, we do not have a control group of patients treated with a different eradication schedule to compare the efficacy of BQT with. However, in a multicenter study in collaboration with Spain, we demonstrated that 14 days CLA-containing concomitant therapy achieved ITT eradication rates of 91.7% (Molina-Infante et al., 2013). Second, in this real-life study, we do not provide information regarding the prevalence of *H. pylori* antimicrobial resistance, which, according to major guidelines (Zagari et al., 2015; Malfertheiner et al., 2017), should searched for only in case of multiple eradication failures. However, we showed previously that in our region the prevalence of *H. pylori* antimicrobial resistance was 26.1% vs. CLA, 33% vs. metronidazole and 7.1% vs. CLA + metronidazole with no *H. pylori* strains resistant to amoxicillin or tetracycline (Molina-Infante et al., 2013).

In summary, this real-life study in an area of high prevalence of *H. pylori* strains with CLA and double resistance shows that an optimized 10 days BQT through the use of the three-in-one capsule is highly effective in eradicating the infection. Moreover, compliance to treatment was optimal with almost all patients taking more than 90% of prescribed medication. Finally, the incidence of TRAEs was as low as 26% with only a minority of patients experiencing severe side effects.

We conclude that optimized single capsule BQT should be recommended as first-line treatment of *H. pylori* infections in areas with high CLA or dual resistance.

## Data Availability

The raw data supporting the conclusions of this article will be made available by the authors, without undue reservation.
